# What Does Pyorrhea Mean to the Work of the Physician?

**Published:** 1917-03

**Authors:** C. M. M’Cauley

**Affiliations:** Dallas, Texas


### What Does Pyorrhea Mean to the Work of the Physician?

BY C. M. M'CAULEY, B. S., D. D. S., DALLAS, TEXAS.

Dr. Osler says that bad teeth are doing more damage to the human race than alcohol. Dr. Mayo has made some very definite statements about the mouth as a focus of infection. As a focus of infection and as a disease-carrier, the condition known as pyorrhea takes rank above dental caries. The reasons are that pyorrhea produces a more unsanitary condition than caries, and the tissues involved are soft tissues from the beginning, while in caries the first stages involve only hard, bloodless tissue. Dental caries is the most prevalent disease known to the human race, and pyorrhea comes in as a close second. Good authority has made the claim that 95 per cent of all people either have pyorrhea or a condition which if allowed to run its course will result in pyorrhea. Like dental caries, pyorrhea is a preventable disease.

### CAUSE, PREVENTION AND CURE.

While there is difference of opinion in the minds of some of our best men as to the cause of some forms of pyorrhea, all seem to agree that the great majority of cases have their origin in local irritation. This irritation is caused by lodgment and fermentation of food debris beneath the free margins of the gum; defective contact between the teeth allowing food to pass through and become wedged between the teeth and to impinge upon the gum. This defective contact is caused by such abnormalities as caries, irregular arrangement of the teeth in the arches, extraction of a tooth allowing others to drift, malformation of a tooth or teeth, malposition of a tooth with reference to the force of mastication, etc.

Pyorrhea usually begins as a small irritated portion of gum tissue and progresses slowly. With the first death of tissue, early in the disease, there is formation of pus in quantity too small to be seen with the unaided eye. It may progress many months, or even many years before pus begins to flow from about the necks of teeth and the teeth become loose. The idea, however, that the patient has no pyorrhea till pus is seen flowing from the necks of teeth and the teeth become loose is erroneous. At the beginning of the disease the lesion is shallow and the gum color deviates but slightly from the normal. As the lesion deepens, due to continuation of the irritant which caused the beginning, the gum shows more evidence of irritation until finally the blood becomes stagnant and the tissues reveal the presence of excessive amount of venous blood. Gums become puffed and tissues die more rapidly. Pus begins to form in quantities sufficient to be squeezed from about the necks of the teeth. This tissue destruction goes on toward the apex of the root, disintegrating alveolar process as well as gum tissue and peridental membrane. When the tooth becomes loosened from this process of death and pus formation, it is because its natural support, namely, the alveolar process and its attached peridental fibers have been destroyed. After these tissues have been destroyed the teeth are forever deprived of their support because these tissues cannot be restored to life. As previously stated, it is often many years between the initial irritation and the time when the tooth loosens. The lesions produced around the teeth are called pockets.

The prevention of pyorrhea is very, very simple. When an irritant appears, remove it before it causes tissue destruction. Also remove the cause of the irritant. The only difficulty about the prevention of pyorrhea is in getting the patient to do his part. The progress of the disease in the first stages is usually painless or nearly so. The patient does not know the necessity for treatment till after the possibility of prevention has passed. It follows then that the remedy is to come with the education of the coming generations. It will take many more generations of suffering and death before the dental profession working alone can bring this about. This teaching must be begun when the child is very young and continued throughout its educational career. Before this can be done its necessity must be fully recognized by everybody who comes into contact with the child in the capacity of teacher. If the medical profession would earnestly join in this educational campaign and work with us till the work is firmly established in our educational system, they would perform a service to the prevention of disease which is second in importance to none which they have ever done.

The cure of pyorrhea consists in the removal of the irritant and the removal of the cause of the irritant and preventing the return of the irritant. With the removal of these the infection goes also, and when the return of these is prevented, recurrence of infection is also prevented. After the supporting fibers and alveolar process around the teeth have been destroyed to a certain degree, all attempts at a cure will prove futile. Charlatans have made good money and are still doing so "curing pyorrhea" in very loose teeth and attaching to them artificial teeth, for which they charge a high price, and boast of a big fee. The safety of the patient's health requires that all very loose teeth, which cannot be treated and placed in a condition free from pus, should be extracted. Those which can be freed of pus and retained in the mouth must be very carefully watched to see that the pus does not return, and thus re-establish the focus of infection.

### EFFECTS OF PYORRHEA UPON THE GENERAL HEALTH.

Pyorrhea as a focus of infection may act in two ways; first by the discharge of pus laden with infection into the mouth and into the alimentary canal; second, by direct absorption of micro-organisms into the circulation. The latter is more likely to take place through the alveolar process when it becomes involved. Bone tissue does not possess the same power to wall off the infectious material as has the soft tissues. The passage of infectious material into the stomach increases as the lesions deepen and as the pus increases in amount. There is no need to enumerate here the different general infections that come from the mouth as a focus.

These have been enumerated again and again by men within your own ranks who speak with authority. Their statements have been based upon results secured by mouth treatment given their patients by the dental profession. For example, during a period of months of the years 1914-15, all patients treated at the University of Minnesota Hospital were placed under care also of the dentist. Twelve per cent of all cases treated at the institution had their trouble definitely traced to mouth conditions as the origin.

Next paper will tell a few things the dental profession is doing in the work of prevention.

---

The Department of Commerce has issued a pamphlet on "The Mortality from Cancer and Other Malignant Humors" in the registration area of the United States, 1914. This monograph presents, in greater detail than has been shown in the annual mortality report of the Bureau of the Census, statistics of deaths which occurred in the registration area during the year 1914 and which were reported as due to cancer and other malignant tumors.

